# Decreased circulating microRNA-21 and microRNA-143 are associated to pulmonary hypertension

**DOI:** 10.55730/1300-0144.5566

**Published:** 2022-10-10

**Authors:** Zekeriya DÜZGÜN, Meral KAYIKÇIOĞLU, Çağdaş AKTAN, Busra BARA, Zuhal EROĞLU, Burcu YAĞMUR, Vildan BOZOK ÇETİNTAŞ, Melike BAYINDIR, Sanem NALBANTGİL, As ı TETİK VARDARLI

**Affiliations:** 1 Department of Medical Biology, Faculty of Medicine, Giresun University, Giresun, Turkey; 2 Department of Cardiology, Faculty of Medicine, Ege University, İzmir, Turkey; 3 Department of Medical Biology, Beykent University School of Medicine, İstanbul, Turkey; 4 Department of Medical Biology, Faculty of Medicine, Ege University, İzmir, Turkey

**Keywords:** Pulmonary hypertension, miRNA, hsa-miR-21-3p, hsa-miR-143-3p, biomarker, vascular remodeling

## Abstract

**Background/aim:**

Pulmonary arterial hypertension (PAH) is characterized by maladaptation of pulmonary vasculature which is leading to right ventricular hypertrophy and heart failure. miRNAs play a crucial role in the regulation of many diseases such as viral infection, cancer, cardiovascular diseases, and pulmonary hypertension (PH). In this study, we aimed to investigate the expression pattern of eight human plasma miRNAs (hsa-miR-21-3p, hsa-miR-143-3p, hsa-miR-138-5p, hsa-miR-145-3p, hsa-miR-190a, hsa-miR-204-3p, hsa-miR-206, hsa-miR-210-3p) in mild-to-severe PH patients and healthy controls.

**Materials and methods:**

miRNAs were extracted from the peripheral plasma of the PH patients (n: 44) and healthy individuals (n: 30) by using the miRNA Isolation Kit. cDNA was synthesized using All in-One First strand cDNA Synthesis Kit. Expression of the human plasma hsa-miR- 21-3p, hsa-miR-143-3p, hsa-miR-138-5p, hsa-miR-145-3p, hsa-miR-190a, hsa-miR-204-3p, hsa-miR-206, hsa-miR-210-3p, and miRNAs were analyzed by qRT-PCR.

**Results:**

According to our results, in PH patients hsa-miR-21-3p and hsa-miR-143-3p expression levels were decreased by 4.7 and 2.3 times, respectively. No significant changes were detected in hsa-miR-138-5p, hsa-miR-145-3p, hsa-miR-190a, hsa-miR-204-3p, hsa- miR-206, and hsa-miR-210-3p expression levels between PH and control groups. In addition, considering the severity of the disease, it was observed that the decrease in miR-138, miR-143, miR-145, miR-190, mir-204, mir-206 and miR-208 expressions was significant in patients with severe PH.

**Conclusion:**

In the early diagnosis of PAH, hsa-miR-21-3p and especially hsa-miR-143-3p in peripheral plasma can be considered as potential biomarkers.

## 1. Introduction

Pulmonary arterial hypertension (PAH) is an important cause of morbidity and mortality, characterized by increased pulmonary vascular resistance and pulmonary artery (PA) pressure (PAP) [1]. PAH affects individuals in a wide range from right heart failure to death by revealing pulmonary vessels by maladaptation. The development of PAH is characterized by structural and specific functional changes mainly associated with the abnormal function of PA endothelial cells (ECs), smooth muscle cells (SMCs), and vascular fibroblasts [2,3]. Although the etiology of PAH development is not yet fully known, it is suggested that a number of biochemical and multiple factors associated with different cell types may play a role in disease formation. Among these factors; a) Excessive vasoconstriction as a result of abnormal function or expression of potassium channels of SMCs, b) Chronic insufficiency in the production of vasodilators such as prostacyclin and nitric oxide, and c) Triggering endothelial dysfunction of vasoconstrictor substances such as endothelin-1 and thromboxane A2 [4].

According to World Health Organization (WHO) classification published in 2019, PAH can develop idiopathically or due to various factors such as drugs and toxins, connective tissue diseases, human immunodeficiency virus, and congenital heart disease. In addition, it has been shown that it may have an autosomal dominantly inherited character due to the germline mutations such as 48G > A and 197G > A occurring in the bone morphogenetic protein (BMP) receptor type 2 (*BMPR2*) gene [5–8]. Numerous factors, including genetic, epigenetic, and environmental factors, play an important role in the pathogenesis of PAH [9]. In recent studies, in addition to *BMPR2*, mutations in Activin A receptor type II-like kinase-1 (*ACVRL1*) and Familial Atrial fibrillation type 7 (*ATFB7*) genes, were shown to cause hereditary PAH and increase the severity of the disease [10,11]. Despite years of research and the development of new treatments, PAH remains a fatal disease. This indicates that there is an urgent need to better understand the pathogenesis of PAH. Common pathological changes such as PA-EC proliferation, PA-SMC proliferation, migration and contraction, inflammation, fibroblast proliferation, activation, and migration are observed in all PAH types [9].

Studies with small, noncoding endogenous RNA molecules (microRNA, miRNA) with a length of 21–25 nucleotides have been shown to be irregular in patients with PAH and experimental pulmonary hypertension (PH). It has been reported that the normalization of several miRNAs including ‎ miR-17~92, miR-204, miR-424, and miR-124 inhibits experimental PH [9,12,13]. These data demonstrate miRNAs are significantly effective with their regulatory role in the development of PH and that circulating miRNAs associated with PH might be potential biomarkers. Rivero and his colleagues have reported that dysregulations in miRNAs such as let-7b-5p, let-7c-5p, miR-1307-3p, miR-185-3p, miR-8084, miR-331-3p, miR-143/145, miR-21, miR-210, miR-138, and miR-210-3p can detriment pulmonary vasculature and cardiac functions. Also, they depicted that these miRNAs can contribute to the development of chronic lung disease (CLD) in the congenital diaphragmatic hernia [14].

Currently, the role of miRNAs in pulmonary vascular remodeling and PH is a research area of interest, however the available data are limited to animal PH models and human PH lung tissue cells. The miRNAs in circulation have not been well characterized in patients with PH. In the present study, we aimed to investigate the expression patterns of circulating miRNAs (miR-21, miR-138, miR-143, miR-145, miR-190, miR-204, miR-206, and miR-210) in PAH patients and compared to healthy individuals.

## 2. Materials and methods

### 2.1. Study population

A total of 44 consecutive adult patients (mean age 46 ±13 years) who were followed at our PAH outpatient’s clinic with the diagnosis of either Group I-PH was enrolled for the study. All patients were aged 18–75 years and fulfilled the diagnostic criteria for precapillary PH [15]. The study population consisted of Group I-PH patients, i.e. PAH those with primary disease process in the pulmonary arteries. Group II-PH (PH due to left heart disease), Group III-PH (PH associated with lung disease), Group IV-PH (patients who had PH due to chronic thromboembolic disease in the pulmonary arteries (CTEPH) and Group V-PH patients were all excluded. Thirty healthy individuals (mean age 63 ±10 years, 17 females, 13 males) served as the control group. We especially chose healthy older individuals to exclude the probability of developing PH in later life in the control subjects as PH is generally a disease of younger patients. History or active malignancy, use of immunomodulatory agents, and active infections were also accepted as exclusion criteria. The study protocol was approved by the Ege University Faculty of Medicine Research Ethics Committee with the decision number of 13-11/92. All participants gave written informed consent.

All patients and controls were evaluated for the association of circulating miRNAs with PH. All PH patients were diagnosed to have PH by right heart catheterization. All patient’s data including WHO functional class, levels of N-terminal of pro-hormone brain natriuretic peptide (NT-proBNP), 6-min walk distance (6MWD), and other laboratory findings were obtained from the patient files which were obtained for routine follow-up during the same period of the blood sampling for miRNAs. While determining the severity of the PH, patients with an mPAP below 35 mmHg were classified to have a mild disease, those between 35 and 70 mmHg as moderate, and those with a greater than 70 mmHg were classified to have a severe PH.

### 2.2. Laboratory analysis

### 2.3. Collection and storage of samples

After peripheral blood sample was collected in 3 mL EDTA tubes from the cases that agreed to participate in the study, the tubes were centrifuged at 1800 rpm at 25 °C for 15 min, and the supernatant (plasma) was placed in cryotubes and stored at −80 °C until miRNA isolation.

### 2.4. Extraction of circulating miRNA

miRNA extraction from 200 μL dissolved plasma was performed in accordance with the QIAGEN^®^ miRNeasy Serum/Plasma kit procedure.

### 2.5. cDNA synthesis

The All-in-One^TM^ miRNA First-Strand cDNA Synthesis Kit was used for cDNA synthesis. Briefly, RNA template was dissolved on ice with 5xPAP/RT Buffer and ddH2O at room temperature. To prepare the miRNA reverse transcriptase reaction solution, a mix was prepared using 2.5 U/μL poly A polymerase, RTase Mix, and 5X PAP/RT buffer in half volume and put in a cold block. The previously prepared 3.5 μL reverse transcriptase reaction solution was mixed with 9 μL isolated total RNA and added to the new 0.2 μL Eppendorf tube. The prepared reaction mixture was incubated at 37 °C for 60 min after a short centrifuge and then incubated at 85 °C for a further 5 min for enzyme inactivation and fixed at 4 °C. The resulting reverse transcription reaction product was made ready for the qPCR step.

### 2.6. Detection of miRNA by qPCR

miRNA detection was performed with 2X All-in-One qPCR Mix and CS-PAM-0617192-96E10 miRNA qPCR array plate. This plate includes hsa-miR-21-3p, hsa-miR-143-3p, hsa-miR-138-5p, hsa-miR-145-3p, hsa-miR-190a, hsa-miR-204-3p, hsa-miR-206, and hsa-miR-210-3p as miRNA primers. RNU6-2 and SNORD44 were preferred as control miRNA primers. The master mix was prepared to mix 5 μL 2X All-in-One qPCR Mix and 4.5 μL ddH2O in a separate tube on the cold block. The cDNAs obtained in the previous process were diluted in half. 0.5 μL cDNA and 9.5 μL master mix were mixed in a separate tube and on a cold block and briefly centrifuged. The miRNA qPCR array embedded in a 96-well plate was taken on a cold block and the cDNA mixture was distributed to this plate in 10 μL. The plate was covered and briefly centrifuged. The plate was placed on LightCycler® 480 Real Time PCR instrument and miRNA expression levels were determined.

### 2.7. Analysis of the results

Analysis of Array data was carried out by ΔΔCT method. In this method, samples and controls were grouped separately and miRNA expression differences among the groups were determined. The p values are calculated based on a Student’s t-test of the replicate 2^(- Delta CT) values for each gene in the control group and treatment groups, and p values less than 0.05 were determined by statistically significant. The significance between average PAP and miRNAs was statistically analyzed with the Kruskal-Wallis test. Descriptive data were presented as percentages or mean ± standard deviation. Differential expressions were assessed using nonparametric Kruskal-Wallis tests across the clinical parameters. Pair-wise comparisons using the Dunn-Bonferroni approach were automatically produced for multiple tests for which the Kruskal-Wallis test was significant using IBM SPSS. Relationship between miRNA expression and clinical parameters was determined using the Spearman correlation coefficient.

## 3. Results

### 3.1. Clinical information and patients’ characteristics

Table 1 presents the clinical characteristics of the study population. When PAH patients were classified according to their diagnoses, most were diagnosed with Eisenmenger physiology followed by idiopathic PAH (iPAH). Most were classified as mild PH according to the severity of the disease (Table 1).

### 3.2. Expression changes of miRNAs in sample of patients diagnosed with PAH

**Figure 1:**
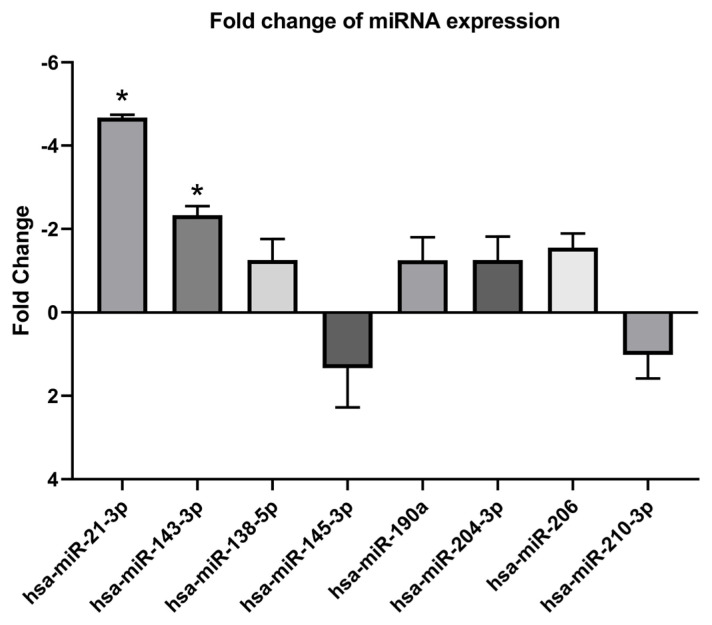
Fold changes of miRNAs compared to the control group. * sign means p < 0.05 according to Student’s t test. Values were normalized according to RNU6-2 and SNORD-44 housekeeping genes.

The expressions of hsa-miR-21-3p and hsa-miR-143-3p in patients’ plasma samples were reduced 4.7 and 2.3 times, respectively. The expression changes of hsa-miR-138-5p, hsa-miR-145-3p, hsa-miR-190a, hsa-miR-204-3p, hsa-miR-206, and hsa-miR-210-3p were not statistically significant between the patients and controls (Table 2) (Figure-1).

**Figure 2:**
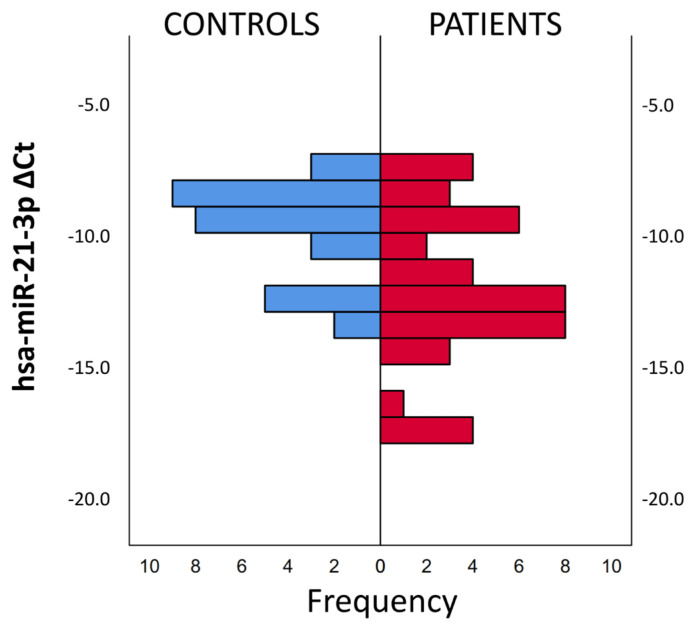
Delta CT frequency ranges of hsa-miR-21-3p expressions by patient group and control group.

The examination of the Delta CT values of miRNA expression changes of the patient and control groups revealed that the hsa-miR-21-3p delta CT values were lower in the PH patients than the control subjects. Lower expressions were observed more frequently in patient group compared to the controls. In the control group, delta CT values accumulated above −10, while in the patient group accumulated below −13 (Figure 2).

**Figure 3:**
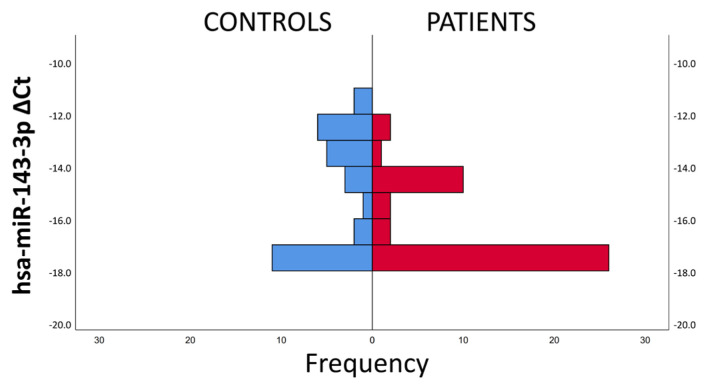
Delta CT frequency ranges of hsa-miR-143-3p expressions by patient group and control group.

In the hsa-miR-143-3p delta CT values, unlike miR-21-3p, accumulation was observed around −17 delta CT values rather than −10 and −13. Similar to miR-21-3p, the frequency of lower delta CT values was more in the patient group, while accumulation was concentrated around −17 delta CT and approximately 3 times more were observed in the patient group (Figure 3).

### 3.3. Evaluation of PH severity according to the miRNA expressions

**Figure 4:**
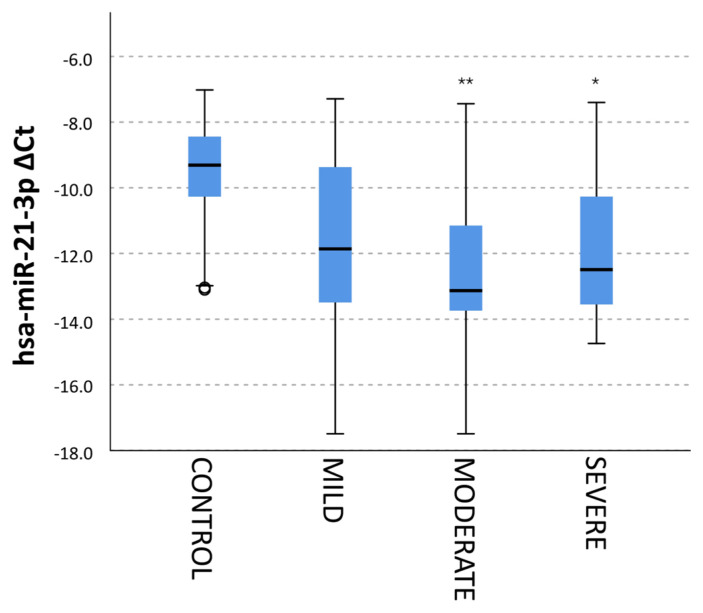
Delta CT values of hsa-miR-21-3p expression according to disease severity. According to the Kruskal-Wallis test, the * sign represents 0.05, and the ** sign symbolizes the 0.01 level of significance.

A comparison of the miRNA expression profiles of the groups showed that the hsa-miR-21-3p expression was lower in the patients than the control subjects, and the expression was at the lowest level, especially in patients with moderately severe PH (Figure 4).

**Figure 5:**
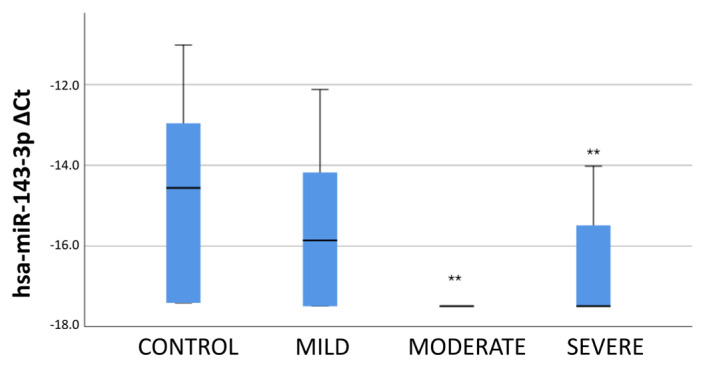
Delta CT values of hsa-miR-143-3p expression according to disease severity. According to the Kruskal-Wallis test, the * sign represents 0.05, and the ** sign symbolizes the 0.01 level of significance.

When the change in the hsa-miR-143-3p expression profile according to the severity of the disease was examined, the expression of miRNA was decreased compared to the control group, similar to the hsa-miR-21-3p and reached the lowest level in the moderately severe PH group (Figure 5).

**Figure 6:**
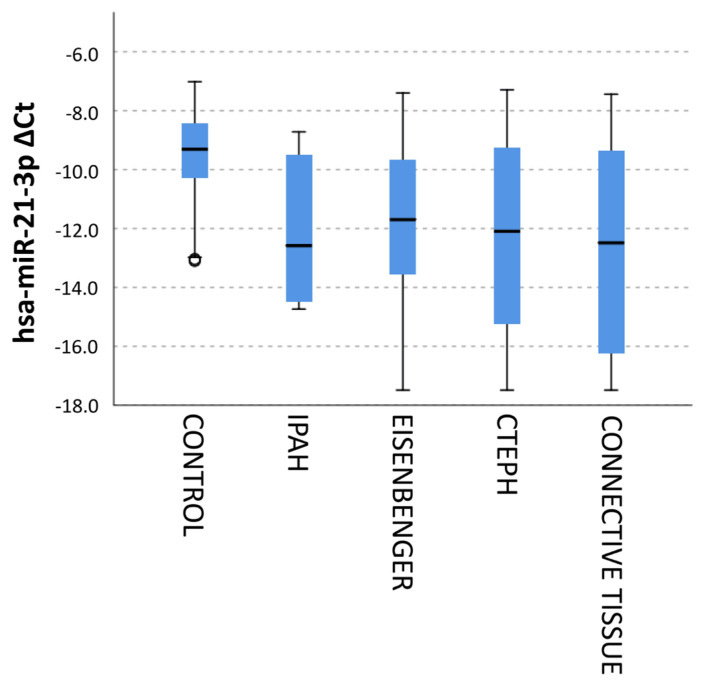
Delta CT values of hsa-miR-21-3p expression according to PAH disease types.

When the expression changes according to PH types were examined, the mean expression change of hsa-miR-21-3p decreased in all disease types compared to the control group (Figure 6).

**Figure 7:**
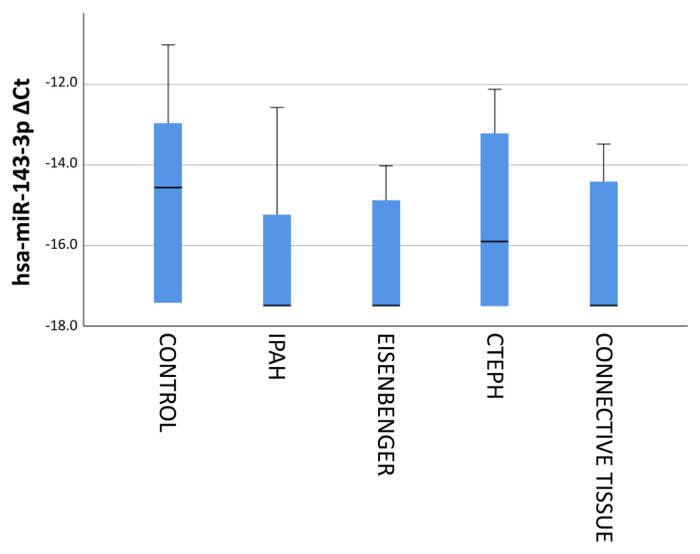
Delta CT values of hsa-miR-143-3p expression according to PAH disease types.

While the delta CT value was above −10 in the control group, and delta CT values below −12 were observed in disease types except patients with Eisenmenger physiology. As the change of hsa-miR-143-3p expression according to disease types was examined, it was observed that the mean delta CT was below −16 except for patients with CTEPH (Figure 7).

## 4. Discussion

In our study, we studied eight different miRNAs that may be potential biomarkers of PH in the plasma of patients with PH and healthy individuals. We observed that the expressions of hsa-miR-21-3p and hsa-miR-143-3p were significantly decreased in patients with PH. The expressions of the rest of the studied miRNAs including hsa-miR-138-5p, hsa-miR-145-3p, hsa-miR-190a, hsa-miR-204-3p, hsa- miR-206, and hsa-miR-210-3p did not differ among the groups. In addition, considering the severity of the disease, it was observed that the decrease in miR-21, miR-138, miR-143, miR-145, miR-190, mir-204, mir-206, and miR-208 expressions were significant in patients with severe PAH.

Currently, miRNAs are popular biomarkers explored in many physiological and pathological conditions as potential targets for novel therapeutic agents [16]. There are several studies exploring different specific miRNAs in PAH or PH either in vivo or in vitro with different methods in the literature. Various miRNAs, including miR-21 and miR-143, have been shown to be associated with PAH in animal models and human lung cells. It has been reported that reduced miR-21 level is associated with the improvement of arterial stiffness in patients with controlled hypertension. Also blocking miR-143-3p has been shown to suppress experimental pulmonary hypertension [17,18]. Wei et al. have reported a significant decrease in the fold changes of miR-1, miR-26a, miR-29c, miR-34b, miR-451, and miR-1246 in 31 patients with PH and 9 healthy control groups. Meanwhile, fold changes of miR-130a, miR-133b, miR-191, miR-204, and miR-208b were increased significantly in PH patients in their study [19]. miR-21 has also been shown to play a central role in the development of both pathological myocardial hypertrophy and myocardial fibrosis in many cellular pathways in recent studies which might be the explanation of our finding to be associated with the severity of the PH [20]. We found that the expression of hsa-miR-21-3p was 4.7 times lower than the control subjects in our study population consisting of PAH and CTEPH patients compared to the control subjects. In the literature, there are few PH studies regarding the expression of miRNA-21-3p with conflicting results. In line with our results, Caruso et al. showed a decrease of miR-21 in human lung tissue and serum in iPAH patients as a confirmatory population for their chronic hypoxia or monocrotaline models in rats [21]. As their manuscript does not provide the number of iPAH patients we can guess to be very few in number as a confirmation population for their rat model. On the contrary, several studies have reported that the increase of miR-21 expression was associated with the development of PAH. Yang et al. have suggested that miR-21 could play an important role in the pathogenesis of hypoxia-dependent pulmonary vascular remodeling and could be a potential biomarker [22]. They found increased expression of miR-21 in distal small arteries in the lungs of hypoxia-exposed mice. They also reported that the sequestration of miR-21, either before or after hypoxia exposure, has diminished the chronic hypoxia-induced PH and attenuated hypoxia-induced pulmonary vascular remodeling. They also have shown high miR-21 expression in the lungs of mice in an experimental pulmonary fibrosis model and also in patients with idiopathic pulmonary fibrosis. In addition, as miR-21 is suggested to be associated with the regulation of pulmonary vascular remodeling in hypoxia-treated mice, blocking miR-21 expression, has been shown to reduce the progression of the restructuring process and the right ventricular hypertrophy-associated vascular remodeling [22]. But all these data represent a completely different mechanism of PH from PAH. However, recent studies showed that miR-21 was upregulated in human PAH PA-ECs and PA-SMCs, human PAH lung tissue, and again hypoxia PH mouse model by microarray method [22–26]. Moreover, inhibition of miR-21 has been shown to prevent hypoxia-induced PH, and therefore, high expression of miR-21 may contribute to the development of PAH in relation to the PA-SMC proliferation and migration [9,27]. Gruning et al. have investigated the miRNA expression changes in the plasma or serum of 31 PH patients with exercise intervention and 21 high altitude-induced PH patients with oxygen intervention using Real-time PCR method. They showed that in samples obtained after exercise intervention, composite miRNA values of miR-22-3p and miR-21-5p/miR-451a and spike RNA were significantly decreased in 65% of the samples [28]. However, Wuttge et al. investigated miRNA expression changes in the plasma of 22 systemic sclerosis-associated PAH patients using a real-time PCR method. They reported that miR-21-5p, miR-130a-3p, miR-150-5p, miR-155-5p, and miR-486-3p showed higher plasma levels in patients with PAH associated with systemic sclerosis compared to patients with systemic sclerosis [29].

miR-143 and miR-145 are abundantly expressed in vascular SMCs and play an important role in their differentiation [30]. In our study, we observed that the expression of hsa-miR-143-3p was decreased by 2.3 times in patients with PH. To the best of our knowledge, our study is the first to evaluate circulating miR-143 in patients with PH. In accordance with our study, Kontaraki et al. showed an almost 2 fold decrease in the expression of miR-143 study in 60 patients with essential hypertension and 29 controls [31]. Deng et al. investigated the transcriptional order of the miR-143/145 cluster and the role of miR-143 in PAH [32]. Their research group identified the promoter region regulating miR-143/145 microRNA expression in PA-SMC and mapped PAH-related signaling pathways including estrogen receptor, liver X factor/retinoic X receptor, transformed growth factor-β (Smads), and hypoxia (hypoxia response element). They observed that miR-143-3p was selectively upregulated during the migration of PA-SMCs compared to miR-143-5p. In addition, contrary to our study, they reported high expression of miR-143-3p in samples from PAH patients [32]. They also showed that miR-143-3p modulates both the cellular and exosome-mediated responses in pulmonary vascular cells and may have an important role in the pathobiology of PAH [32]. In another study, Wang et al. demonstrated a positive correlation between hypoxia-dependent PH and Hypoxia-inducible factor-1 (HIF-1) expression in newborn rats [33]. Tang et al. showed that miR-143 inhibited HIF1 which acts as an important transcription factor regulating cell viability under hypoxic conditions [34,35]. This may be due to polymorphic variations between populations or characteristic differences of PH between selected patient groups.

Though we could not find any significant change in circulating hsa-miR-138-5p, hsa-miR-145-3p, hsa-miR-190a, hsa-miR-204-3p, hsa-miR-206, and hsa-miR-210-3p except PH severity in the present study, various studies have been reported that these miRNAs could play a role in pathogeneses of PAH or PH. In a study, PA-SMC was isolated from healthy controls and PAH patients, and in vivo and in vitro effects of miR-138 expression were investigated. As a result, high miR-138 expression was suggested to directly regulate mitochondrial calcium uniporter (MCU) in PAH. Nebulized anti-miRs against miR-138 have been shown to restore MCU expression, reduce cell proliferation, and regress PAH in the monocrotaline model [36]. Another study has suggested that miR-138 promotes the proliferation of PA-SMCs and promotes suppressed mitochondrial depolarization of SMCs by targeting potassium channel subfamily K member 3 (TASK-1) [37]. Though we did not find a change, our study is the first to explore the circulating levels of miR-138 in patients with PH.

Though did not reach a statistically significant level, the expression of miR-145 was increased by 34% in our study. Similarly, Sarrion et al. have examined miRNA expression changes in serum obtained from 12 well-characterized iPAH patients by microarray method and observed that miR-145 was increased 3.21 times compared to the control group [38]. Caruso et al. investigated the effect of miR-145 on PAH development, and they localized miR-145 to SMCs in the rat lungs and exposed it to hypoxia and showed that the expression of miR-145 was increased. The same group also showed that miR-145 deficiency and anti-miR-mediated reduction provide significant protection against PAH development [39].

For miR-190 expression, we observed no difference between the patient and control groups. However, several studies have suggested that miR-190 might be associated with PAH. Li et al. used synthetic miRNA to mimic the miR-190 increase in hypoxic conditions and investigated the effects of miR-190 in PA and demonstrated that synthetic miR-190 significantly increased vasoconstriction responses to phenylephrine (PE) and KCl [40]. In another study, Blissenbach and colleagues have shown a significant correlation between circulating miR-190 and systolic PAP in a model of hypoxia-related PH in humans exposed to extreme altitude in 40 healthy volunteers [41]. Jiang et al. have measured circulating levels of miR-190a-5p by quantitative real-time PCR with plasma samples taken from 73 patients with chronic obstructive pulmonary disease (COPD)-PH and 32 healthy controls. They showed that the level of miR-190a-5p was significantly higher in PH patients compared to healthy controls. [42]. However, as COPD-associated PH (Group-3 PH) physiopathology differs from PAH we have excluded these patients from our study. Therefore, our study is the first to evaluate circulating miR-190 in patients with PAH.

miR-204 is accepted as a promising miRNA in the field of PAH [16]. In our study, a 21% decrease was observed in miR-204 expression. Also, Sarrion et al. have observed that the circulating levels of miR-204 decreased 4.67 times compared to the control group well-characterized iPAH patients by microarray method [38]. Similarly, Courboulin et al. showed reduced miR-204 expression might be associated with PAH in 13 patients with PAH. They reported that STAT3 activation suppresses miR-204 expression and miR-204 directly targets SHP2 expression, therefore, by regulating SHP2 up and miR-204 down, it activates Src kinase and active T cell nuclear factor (NFAT) [12]. Li et al. measured plasma miR-204 expression one h before and 7 days after cardiac surgery in 52 children with congenital heart disease (left to right shunt). They observed that the circulating levels of miR-204 were lower in children with PH (n = 39) compared to those without PH (n = 13). In these children, miR-204 expression tended to decrease after surgery in those with or without PH. They also showed that systolic PAP was negatively correlated with miR-204 expression in these patients [43].

In our study, the 36% decrease in the expression of miR-206 was compatible with the study of Jin and colleagues conducted on patients with left heart disease (LHD). They found that the level of miR-206 was significantly decreased in those with LHD-associated PH compared with that of the LHD and healthy control groups. Moreover, miR-206 levels were negatively correlated with systolic PAP [44]. We have excluded LHD-associated PH due to the pathogenetic difference between PAH and those with LHD-associated PH.

miR-210 plasma expression did not significantly differ between our patient and control groups. However, several studies have reported that miR-210 might be associated with PAH. Researchers have reported possible interactions between miR-210 and mitogen-activated protein kinase phosphatase 1 (MKP-1) and its effects on cell proliferation in hypoxic human PA-SMCs. They observed that miR-210 increased significantly in MKP-1, mRNA, and protein expression, as well as cultured human PA-SMCs exposed to hypoxia with 1% O_2_ for 48 h. Overexpression of miR-210 was also suggested to prevent hypoxia-induced MKP-1 expression, which has no effect on cell proliferation [45].

Various studies have shown that contradictory conclusions have been reached as to whether the decrease or increase in the expression of some miRNAs may have an impact on PH and/or PAH. Although our study generally complies with the literature, some miRNAs such as miR-143 appear to have conflicting results. This discordance in the literature is probably due to the differences in methodologies and also the samples worked on. In general, most of the studies are conducted in vascular cells of animal models of either monocrotaline or hypoxia-induced PH. However, these two models are completely different, i.e. the first generates a strong pulmonary inflammation by targeting the PA-ECs associated with monocyte recruitment, similar to that observed in human iPAH [16]. However, the latter, persistent hypoxia, affects all 3 layers of the pulmonary arteriolar wall associated with significant hypertrophy of PA-SMCs and less inflammation [16]. The milieu studied for analysis of the miRNA could be important in the results. Most of the studies in the literature are conducted in lung vascular cells (PA-SCM or PA-ECs) in animals and to a lesser degree in humans. Differently, we studied the circulating miRNAs which is another way of analyzing miRNAs. There are only a few studies measuring the circulating miRNAs in PH in the literature. To the best of our knowledge, our study is the first to measure circulating miR-21, miR-143, miR-138, miR-145, miR-190a, miR-206, and miR-210 in patients with PAH in the literature. The suggested roles of the miRNAs that we studied are proliferation for miR-21, miR-143, miR-138, miR-145, and miR-210, vasoconstriction for miR-190a, vasoconstriction and DNA damage for miR-204 and finally proliferation and angiogenesis miR-206 [16]. However, most of these roles are generated from tissue analysis of animal or human PH models.

## 5. Study limitations

The small sample size could be regarded as a limitation of our study, however, 44 patients with PAH or CETPH is a good sample size for a rare disease from a single center. Including CETPH patients should not be considered as a limitation, though the triggering pathology is thrombus, the same histopathologic changes develop both in PAH and CTEPH. It is well known that noncardiac conditions, such as cancer, infection, and drug use may affect miRNA expression. Therefore, to overcome this limitation we excluded patients with a past or active cancer history, active infections, and those on immunomodulator therapy. The older age of the healthy control group could be regarded as a limitation, however, we specifically preferred to enroll older aged individuals to exclude the probability of developing PH in later life in the control subjects as PH is generally a disease of younger patients. But the major strength of our study is the evaluation of the circulating levels of miRNAs in patients with PH since the available data in the literature is almost generated from different experimental models and PH human tissues. Moreover, most data are covering the hypoxia-induced animal models or patient populations such as pulmonary fibrosis or COPD that were all excluded from our study.

## 6. Conclusions

This cross-sectional evaluation of 44 PH patients with either PAH or CETPH showed that the expression of circulating miR-21 and miRNA-143 are significantly higher than the control subjects. However, there were no significant difference in circulating levels of hsa-miR-138-5p, hsa-miR-145-3p, hsa-miR-190a, hsa-miR-204-3p, hsa-miR-206, and hsa-miR-210-3p except compared PAH severity in those with PH compared to healthy controls. Though we could not find difference in the latter 6 miRNAs, our results will serve to further figure out the role of these miRNAs as biomarkers of diagnosis and/or therapy in PH since our study is the first to quantify the circulating levels of the most of these miRNAs. Further studies evaluating the circulating levels of miRNAs in different patients’ groups with different PH etiologies are needed to clarify their promising role in the diagnosis, prognosis, and also treatment of PH.

## Figures and Tables

**Table 1:** Clinical characteristics of the study population.

	PH patients (n = 44)	Controls (n = 30)
Age (mean ± SD), years	46 ± 13	63 ± 10
Sex female, n (%)	32 (73%)	17 (56.7)
BMI (mean ± SD)	24.58 ± 6.42	27.58 ± 4.27
NT-proBNP levels (pg/mL) (mean ± SD).	731 ± 969	
6-min walk distance (m) (mean ± SD)	423 ± 89	
Diagnosis, (n)		
iPAH	10 (22.7)	
EISENMENGER	25 (56.8)	
CTEPH	4 (9.1)	
Connective tissue disease	5 (11.4)	
PH severity		
Mild	6 (13.6)	
Moderate	25 (56.8)	
Severe	13 (29.5)	

Definition of abbreviations: BMI = Body Mass Index.

iPAH = Idiopathic Pulmonary Artery Hypertension.

CTEPH = Chronic thromboembolic pulmonary hypertension

PH: Pulmonary Hypertension

NT-proBNP: N-terminal prohormone of brain natriuretic peptide

**Table 2:** Fold changes of miRNAs according to the control group.

miRNAs	Fold change
hsa-miR-21-3p	−4.6723
hsa-miR-143-3p	−2.3333
hsa-miR-138-5p	−1.2615
hsa-miR-145-3p	1.3364
hsa-miR-190a	−1.2523
hsa-miR-204-3p	−1.2601
hsa-miR-206	−1.557
hsa-miR-210-3p	1.0158

## References

[1] Barst RJ McGoon MD Elliott CG Foreman AJ Miller DP Survival in childhood pulmonary arterial hypertension: Insights from the registry to evaluate early and long-term pulmonary arterial hypertension disease management Circulation 2012 125 1 113 122 10.1161/CIRCULATIONAHA.111.026591 22086881

[2] Kayıkçıoğlu M Pulmoner hipertansiyonda etiyopatogenez: İnflamasyon, vasküler yeniden şekillenme Anadolu Kardiyoloji Dergisi 2010 10 5 8 10.5152/akd.2010.113 (in Turkish) 20819761

[3] Bienertova-Vasku J Novak J Vasku A MicroRNAs in pulmonary arterial hypertension: pathogenesis, diagnosis and treatment Journal of the American Society of Hypertension: JASH 2015 9 3 221 234 10.1016/j.jash.2014.12.011 25660363

[4] Nakanishi N 2009 ESC/ERS pulmonary hypertension guidelines and connective tissue disease Allergology International 2011 60 4 419 424 10.2332/allergolint.11-RAI-0362 22015568

[5] Lane KB Machado RD Pauciulo MW Thomson JR Phillips JA Heterozygous germline mutations in BMPR2, encoding a TGF-beta receptor, cause familial primary pulmonary hypertension Nature genetics 2000 26 1 81 84 10.1038/79226 10973254

[6] Sztrymf B Coulet F Girerd B Yaici A Jais X Clinical outcomes of pulmonary arterial hypertension in carriers of BMPR2 mutation American Journal of Respiratory and Critical Care Medicine 2008 177 12 1377 1383 10.1164/rccm.200712-1807OC 18356561

[7] Simonneau G Montani D Celermajer DS Denton CP Gatzoulis MA Haemodynamic definitions and updated clinical classification of pulmonary hypertension The European respiratory journal 2019 53 1 10.1183/13993003.01913-2018 PMC6351336 30545968

[8] Mutlu Z Kayıkçıoğlu M Nalbantgil S Vuran Ö Kemal H Sequencing of mutations in the serine/threonine kinase domain of the bone morphogenetic protein receptor type 2 gene causing pulmonary arterial hypertension Anatolian Journal of Cardiology 2016 16 7 491 496 10.5152/AnatolJCardiol.2015.6297 26645265 PMC5331396

[9] Zhou G Chen T Raj JU MicroRNAs in Pulmonary Arterial Hypertension American Journal of Respiratory Cell and Molecular Biology 2015 52 2 139 151 10.1165/rcmb.2014-0166TR 25192340 PMC4370247

[10] Girerd B Montani D Coulet F Sztrymf B Yaici A Clinical outcomes of pulmonary arterial hypertension in patients carrying an ACVRL1 (ALK1) mutation American journal of respiratory and critical care medicine 2010 181 8 851 61 10.1164/RCCM.200908-1284OC 20056902

[11] Pousada G Baloira A Vilariño C Cifrian JM Valverde D Novel mutations in BMPR2, ACVRL1 and KCNA5 genes and hemodynamic parameters in patients with pulmonary arterial hypertension PLoS ONE 2014 9 6 e100261 10.1371/journal.pone.0100261 24936649 PMC4061078

[12] Courboulin A Paulin R Giguère NJ Saksouk N Perreault T Role for miR-204 in human pulmonary arterial hypertension Journal of Experimental Medicine 2011 208 3 535 548 10.1084/jem.20101812 21321078 PMC3058572

[13] Jalali S Ramanathan GK Parthasarathy PT Aljubran S Galam L Mir-206 regulates pulmonary artery smooth muscle cell proliferation and differentiation PloS one 2012 7 10 e46808 10.1371/journal.pone.0046808 23071643 PMC3468623

[14] Herrera-Rivero M Zhang R Heilmann-Heimbach S Mueller A Bagci S Circulating microRNAs are associated with Pulmonary Hypertension and Development of Chronic Lung Disease in Congenital Diaphragmatic Hernia Scientific Reports 2018 8 1 1 11 10.1038/s41598-018-29153-8 30013141 PMC6048121

[15] Galiè N Humbert M Vachiery JL Gibbs S Lang I 2015 ESC/ERS Guidelines for the diagnosis and treatment of pulmonary hypertension European Heart Journal 2016 37 1 67 119 10.1093/eurheartj/ehv317 26320113

[16] Santos-Ferreira CA Abreu MT Marques CI Gonçalves LM Baptista R Micro-RNA Analysis in Pulmonary Arterial Hypertension: Current Knowledge and Challenges JACC: Basic to Translational Science 2020 5 11 1149 1162 33294743 10.1016/j.jacbts.2020.07.008 PMC7691282

[17] Miao C Chang J Zhang G Recent research progress of microRNAs in hypertension pathogenesis, with a focus on the roles of miRNAs in pulmonary arterial hypertension Molecular Biology Reports 2018 45 6 2883 2896 10.1007/s11033-018-4335-0 30298350

[18] Wu D Talbot CC Liu Q Jing ZC Damico RL Identifying microRNAs targeting Wnt/β-catenin pathway in end-stage idiopathic pulmonary arterial hypertension Journal of molecular medicine 2016 94 8 875 885 10.1007/S00109-016-1426-Z 27188753 PMC4956511

[19] Wei C Henderson H Spradley C Li L Kim I-K Circulating miRNAs as Potential Marker for Pulmonary Hypertension PLoS ONE 2013 8 5 e64396 10.1371/journal.pone.0064396 23717609 PMC3662705

[20] Ben-Nun D Buja LM Fuentes F Prevention of heart failure with preserved ejection fraction (HFpEF): reexamining microRNA-21 inhibition in the era of oligonucleotide-based therapeutics Cardiovascular Pathology 2020 1 49 1 11 10.1016/j.carpath.2020.107243 32629211

[21] Caruso P MacLean MR Khanin R McClure J Soon E Dynamic changes in lung microRNA profiles during the development of pulmonary hypertension due to chronic hypoxia and monocrotaline Arteriosclerosis, thrombosis, and vascular biology 2010 30 4 716 723 10.1161/ATVBAHA.109.202028 20110569

[22] Yang S Banerjee S Freitas A de Cui H Xie N miR-21 regulates chronic hypoxia-induced pulmonary vascular remodeling American Journal of Physiology-Lung Cellular and Molecular Physiology 2012 302 6 L521 L529 10.1152/ajplung.00316.2011 22227207 PMC3311531

[23] Iannone L Zhao L Dubois O Duluc L Rhodes CJ miR-21/DDAH1 pathway regulates pulmonary vascular responses to hypoxia Biochemical Journal 2014 462 1 103 112 10.1042/BJ20140486 24895913

[24] Zhu B Gong Y Yan G Wang D Qiao Y Down-regulation of lncRNA MEG3 promotes hypoxia-induced human pulmonary artery smooth muscle cell proliferation and migration via repressing PTEN by sponging miR-21 Biochemical and Biophysical Research Communications 2018 495 3 2125 2132 10.1016/j.bbrc.2017.11.185 29198701

[25] Green DE Murphy TC Kang B-Y Searles CD Hart CM PPARγ Ligands Attenuate Hypoxia-Induced Proliferation in Human Pulmonary Artery Smooth Muscle Cells through Modulation of MicroRNA-21 West J PLOS ONE 2015 10 7 e0133391 10.1371/journal.pone.0133391 PMC4514882 26208095

[26] Parikh VN Jin RC Rabello S Gulbahce N White K MicroRNA-21 integrates pathogenic signaling to control pulmonary hypertension: Results of a network bioinformatics approach Circulation 2012 125 12 1520 1532 10.1161/CIRCULATIONAHA.111.060269 22371328 PMC3353408

[27] Sarkar J Gou D Turaka P Viktorova E Ramchandran R MicroRNA-21 plays a role in hypoxia-mediated pulmonary artery smooth muscle cell proliferation and migration American journal of physiology Lung cellular and molecular physiology 2010 299 6 L861 L871 10.1152/ajplung.00201.2010 20693317 PMC3006273

[28] Grunig G Eichstaedt CA Verweyen J Durmus N Saxer S Circulating MicroRNA markers for pulmonary hypertension in supervised exercise intervention and nightly oxygen intervention Frontiers in Physiology 2018 1 9 1 13 10.3389/fphys.2018.00955 PMC6068281 30090067

[29] Wuttge DM Carlsen AL Teku G Wildt M Rådegran G Circulating plasma microRNAs in systemic sclerosis-associated pulmonary arterial hypertension Rheumatology 2022 61 1 309 318 10.1093/rheumatology/keab300 PMC8742821 33784391

[30] Boettger T Beetz N Kostin S Schneider J Krüger M Acquisition of the contractile phenotype by murine arterial smooth muscle cells depends on the Mir143/145 gene cluster The Journal of clinical investigation 2009 119 9 2634 2647 10.1172/JCI38864 19690389 PMC2735940

[31] Kontaraki JE Marketou ME Zacharis EA Parthenakis FI Vardas PE Differential expression of vascular smooth muscle-modulating microRNAs in human peripheral blood mononuclear cells: novel targets in essential hypertension Journal of Human Hypertension 2014 28 8 510 516 10.1038/jhh.2013.117 24284386

[32] Deng L Blanco FJ Stevens H Lu R Caudrillier A MicroRNA-143 Activation Regulates Smooth Muscle and Endothelial Cell Crosstalk in Pulmonary Arterial Hypertension Circulation Research 2015 117 10 870 883 10.1161/CIRCRESAHA.115.306806 26311719 PMC4620852

[33] Wang H Paulsen MJ Hironaka CE Shin HS Farry JM Natural heart regeneration in a neonatal rat myocardial infarction model Cells 2020 9 1 229 10.3390/CELLS9010229 31963369 PMC7017245

[34] Wang L Zhou Y Li MX Zhu YP Expression of hypoxia-inducible factor-1α, endothelin-1 and adrenomedullin in newborn rats with hypoxia-induced pulmonary hypertension Experimental and Therapeutic Medicine 2014 8 1 335 339 10.3892/etm.2014.1728 24944643 PMC4061228

[35] Tang B ming Tang M mei Xu Q lu Guo J Xuan L MicroRNA-143-5p modulates pulmonary artery smooth muscle cells functions in hypoxic pulmonary hypertension through targeting HIF-1α Journal of Biosciences 2020 45 1 1 9 10.1007/s12038-020-9992-1 32098916

[36] Hong Z Chen K-H DasGupta A Potus F Dunham-Snary K MicroRNA-138 and MicroRNA-25 Down-regulate Mitochondrial Calcium Uniporter, Causing the Pulmonary Arterial Hypertension Cancer Phenotype American journal of respiratory and critical care medicine 2017 195 4 515 529 10.1164/rccm.201604-0814OC 27648837 PMC5378421

[37] Liu J-J Zhang H Xing F Tang B Wu S-L MicroRNA-138 promotes proliferation and suppresses mitochondrial depolarization in human pulmonary artery smooth muscle cells through targeting TASK-1 Molecular medicine reports 2018 17 2 3021 3027 10.3892/mmr.2017.8200 29257242 PMC5783522

[38] Sarrion I Milian L Juan G Ramon M Furest I Role of circulating miRNAs as biomarkers in idiopathic pulmonary arterial hypertension: Possible relevance of miR-23a Oxidative Medicine and Cellular Longevity 2015 1 2015 1 10 10.1155/2015/792846 PMC4357130 25815108

[39] Caruso P Dempsie Y Stevens HC McDonald RA Long L A role for miR-145 in pulmonary arterial hypertension: evidence from mouse models and patient samples Circulation research 2012 111 3 290 300 10.1161/CIRCRESAHA.112.267591 22715469

[40] Li S-S Ran Y-J Zhang D-D Li S-Z Zhu D MicroRNA-190 regulates hypoxic pulmonary vasoconstriction by targeting a voltage-gated K^+^ channel in arterial smooth muscle cells Journal of cellular biochemistry 2014 115 6 1196 1205 10.1002/jcb.24771 24446351

[41] Blissenbach B Nakas CT Krönke M Geiser T Merz TM Hypoxia-induced changes in plasma micro-RNAs correlate with pulmonary artery pressure at high altitude 2018 314 1 L157 L164 10.1152/ajplung.00146.2017 28971974

[42] Jiang J Xia Y Liang Y Yang M Zeng W MiR-190a-5p participates in the regulation of hypoxia-induced pulmonary hypertension by targeting KLF15 and can serve as a biomarker of diagnosis and prognosis in chronic obstructive pulmonary disease complicated with pulmonary hypertension International Journal of COPD 2018 1 13 3777 3790 10.2147/COPD.S182504 PMC6251363 30538440

[43] Li X Xiang D Shu Y Hu K Zhang Y MicroRNA-204 as an indicator of severity of pulmonary hypertension in children with congenital heart disease complicated with pulmonary hypertension Medical Science Monitor 2019 25 10173 10179 10.12659/MSM.917662 31887731 PMC6951116

[44] Jin P Gu W Lai Y Zheng W Zhou Q The Circulating MicroRNA-206 Level Predicts the Severity of Pulmonary Hypertension in Patients with Left Heart Diseases Cellular Physiology and Biochemistry 2017 41 6 2150 2160 10.1159/000475569 28554172

[45] Jin Y Pang T Nelin LD Wang W Wang Y MKP-1 is a target of miR-210 and mediate the negative regulation of miR-210 inhibitor on hypoxic hPASMC proliferation Cell Biology International 2015 39 1 113 120 10.1002/cbin.10339 25044272

